# A Case of Bradycardia, Renal Failure, Atrioventricular Nodal Blockade, Shock, and Hyperkalaemia (BRASH) Syndrome in an Elderly Male and Its Management: A Case Report and Literature Review

**DOI:** 10.7759/cureus.49489

**Published:** 2023-11-27

**Authors:** Akbar Hussain, Nazneen Ahmed, Stanley Marlowe, Jonathan Piercy, Sai S Kommineni

**Affiliations:** 1 Internal Medicine, Appalachian Regional Healthcare, Harlan, USA

**Keywords:** shock, bradycardia, hyperkalemia, av nodal blockage, brash syndrome

## Abstract

BRASH syndrome, characterized by bradycardia, renal dysfunction, atrioventricular (AV) nodal blockage, shock, and hyperkalemia, is a rare but potentially life-threatening condition resulting from the interplay between AV nodal blockers and hyperkalemia. This complex syndrome poses significant challenges in diagnosis and management, with patients often presenting with bradycardia and high potassium levels. This case report highlights the need for increased awareness of BRASH syndrome, especially in an aging population and evolving cardiovascular treatments. Early recognition and a comprehensive, multidisciplinary approach are crucial for improving outcomes in affected patients.

## Introduction

BRASH syndrome is a condition where a sequence of events involving atrioventricular (AV) nodal blockers and impaired kidney function leads to significant bradycardia and elevated blood potassium levels. The name itself reflects its primary symptoms: bradycardia, renal dysfunction, AV nodal blockage, shock, and hyperkalemia [1]. This combination sets off a chain reaction, causing a range of issues that impact the heart rhythm, kidney function, and potassium levels, all interconnected in this complex syndrome.

Beta-blockers and calcium channel blockers have been used extensively since the 1960s to treat illnesses, such as coronary artery disease, hypertension, and atrial fibrillation. Both drug classes affect the AV node, which lowers the heart rate. Like many medications, AV nodal blockers may cause side effects. The most frequent ones are fatigue, drowsiness, disturbed sleep, and shortness of breath. However, it is crucial to remember that BRASH syndrome is a relatively under-recognized condition that, if not promptly recognized and treated, can pose serious health hazards, ultimately leading to cardiovascular collapse and multi-organ failure [1]. Due to the rising prevalence of aging-related health problems and the introduction of drugs intended to affect heart function, this syndrome represents a rare medical disease that is increasingly being observed as a leading cause of severe bradycardia. The complex interaction between renal failure and AV nodal obstruction results in a self-sustaining loop of severe bradycardia and high blood potassium levels. It is hypothesized that this illness, despite being common, frequently goes overlooked and, as a result, is incorrectly identified in intensive care units (ICUs) [2]. Even though the precise prevalence of BRASH syndrome is not yet fully understood, the combination of an aging population and the adoption of more proactive preventive healthcare measures, such as stricter blood pressure control, highlights a greater need of knowledge of the syndrome's distinctive symptoms and methods of treatment [3]. There have only been a few cases reported since it was recognized as a clinical illness, and much more has to be understood about how this sickness manifests itself [2,4].

We present a case of a 77-year-old male who had bilateral lower extremity (LE) edema and later developed sudden scrotal and penile swelling, along with various complications, prompting consideration of BRASH syndrome.

## Case presentation

We present a challenging case of a 77-year-old male patient with initial complaint of bilateral LE edema, a symptom he had been experiencing for four months. However, over two days, he developed a sudden onset of scrotal and penile swelling, accompanied by increased urinary frequency and decreased urinary output. The patient has past medical history of diabetes mellitus, hypertension, and chronic obstructive pulmonary disease and underwent right partial pulmonary lobectomy.

Upon admission, the patient's vital signs were as follows: his temperature was 97.9°F, pulse rate was 50 beats per minute, respiration rate 10 breaths per minute, blood pressure was elevated at 169/94 mmHg, and his pulse oximetry reading was 93% on 6 L per minute of oxygen delivered via a nasal cannula. The initial reading shows a blood urea nitrogen (BUN) of 67 mg/dL and a creatinine of 2.75 mg/dL, while the subsequent reading demonstrates a BUN of 53 mg/dL and a creatinine of 2.05 mg/dL, indicative of acute kidney injury (AKI). The urinalysis reveals no red blood cells, 0-2 white blood cells, no squamous epithelial cells, and no bacteria observed per high-power-field examination. The patient's lab values are shown in Table 1.

**Table 1:** Patient lab values at hospital admission.

Test	Result	Normal Range
N-terminal pro-brain natriuretic peptide (NT-proBNP)	3,928 pg/ml	0-450 pg/ml
Aspartate aminotransferase (AST)	28 U/L	15-37 U/L
Alanine aminotransferase (ALT)	7 U/L	16-63 U/L
Alkaline phosphatase	157 U/L	46-116 U/L
Total bilirubin	0.70 mg/dL	0.20-1.00 mg/dL
Sodium	135 mmol/L	136-145 mmol/L
Potassium	5.3 mmol/L	3.5-5.1 mmol/L
Chloride	104 mmol/L	98-107 mmol/L
Carbon dioxide (CO_2_)	24.2 mmol/L	21.0-32 mmol/L
Blood urea nitrogen (BUN)	53 mg/dL	7.0-18.0 mg/dL
Creatinine	2.05 mg/dL	0.70-1.30 mg/dL
Glucose	104 mg/dL	70-99 mg/dL
Thyroid function test (TFT)		
Thyroid-stimulating hormone (TSH)	2.928 mlU/mL	0.358-3.740 mlU/mL
Free T4	1.03 ng/dL	0.76-1.46 ng/dL

**Figure 1:**
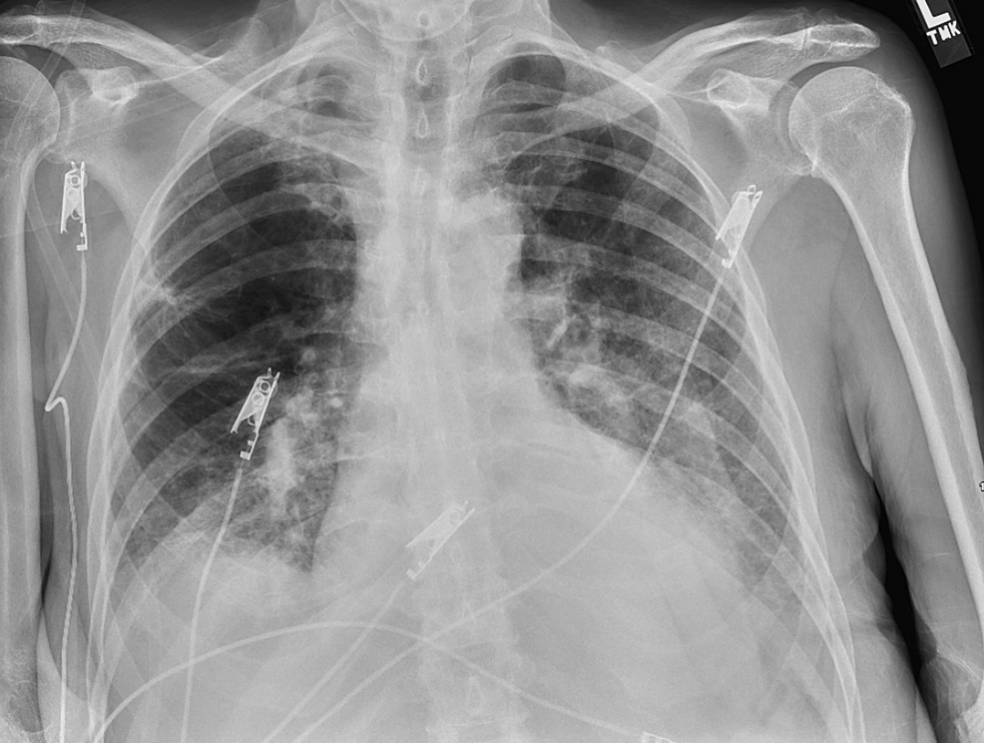
Chest X-ray showing interstitial thickening with ground-glass density in both lower lobes.

During the patient's hospitalization, a range of medical challenges surfaced, adding layers of complexity to the case. These challenges included respiratory failure accompanied by hypoxia, instances of bradycardia with heart rates consistently below 60 beats per minute, congestive heart failure marked by a significantly reduced ejection fraction, episodes of hypotension, the onset of AKI, elevated potassium levels resulting in hyperkalemia, urinary retention concerns, and observations of seizure-like activities. Bradycardia is characterized by heart rates consistently below 60 beats per minute, presented as either mild bradycardia (with heart rates between 50 and 60), or, in more severe cases, associated with hemodynamic compromise. The specific rhythm, such as sinus bradycardia or AV block, and its potential impact on symptoms or hemodynamic status were under scrutiny. The patient's chest X-ray is shown in Figure 1.

The patient's medical assessment and plan encompass various health concerns. Initially, the patient experienced respiratory failure with hypoxia, which was alleviated with the use of a non-rebreather mask. Suspecting a pulmonary embolism due to worsening AKI, heparin treatment was initiated. Bradycardia was observed on telemetry, leading to adjustments in carvedilol dosage, and pacer pads were prepared but ultimately not needed. In the context of congestive heart failure, cardiology was consulted, resulting in modifications in medication, including spironolactone, furosemide, and carvedilol, with continuous monitoring of sinus rhythm and blood pressure. The patient also experienced hypotension, with episodes of dizziness during exertion, prompting the continuation of midodrine. In addition, AKI necessitated measures and consultation with nephrology. Hyperkalemia were managed through insulin, hypertonic glucose, sodium bicarbonate, and calcium gluconate administration. Other concerns included urinary retention, seizure-like activity, scrotal swelling, and lower-extremity edema, all of which are being closely monitored and managed. Further follow-up and outpatient care will be essential to monitor the patient's progress and long-term health. The patient responded well to the treatment given.

## Discussion

Patients with BRASH syndrome can present in a wide range of clinical ways, from asymptomatic bradycardia to severe instances requiring hemodialysis and vasopressors owing to cardiogenic shock. Nevertheless, all patients with this disease will have noticeable bradycardia, regardless of the degree. Patients with this syndrome typically have a history of recent gastrointestinal disorders, dehydration, medication changes, or other circumstances that are known to raise the risk of acute kidney damage. This condition is frequently brought on by diminished renal perfusion. The dosage used does not impact the effectiveness of AV nodal blockers in managing this condition [5].

The primary underlying pathophysiologic characteristic of this illness is an interplay between AV nodal blockers and hyperkalemia that results in bradycardia, as shown in Figure 1 [4, 6]. This combination can result in severe bradycardia in BRASH syndrome patients even when potassium levels are about 5.0 mEq/L, a range that is generally not associated with major arrhythmias [1]. Because there are several components to this condition, doctors typically focus on treating the most noticeable one rather than treating the syndrome as a whole while coming up with resuscitative measures. It is critical to understand that this is a syndrome and not an underlying cause, describing a collection of symptoms. Both bradycardia and hyperkalemia might present conditions that necessitate prompt medical attention. Although supportive treatment often improves the majority of patients, it is crucial to follow the approved standard course of action for treating hyperkalemia.

**Figure 2:**
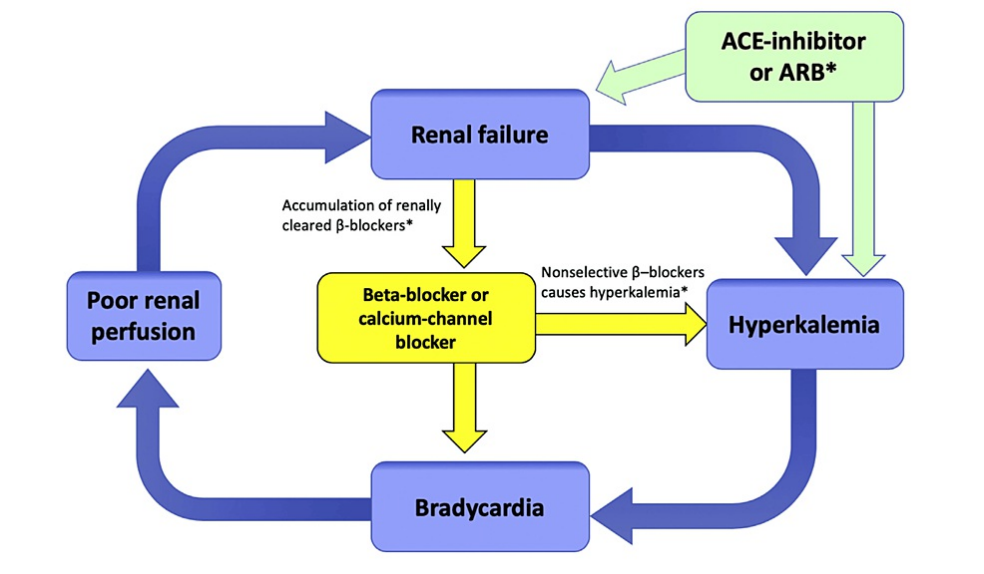
BRASH (bradycardia, renal failure, AV blockade, shock, and hyperkalemia) syndrome pathophysiology. Reference: Farkas et al. [6] * indicates components that are not required but may contribute in some cases. ACE: angiotensin-converting enzyme; ARB: angiotensin-receptor blocker Copyright/license: This figure has been adapted from an open-source article [6] distributed under the terms and conditions of the Creative Commons CC-BY-NC-ND license.

Initiating intravenous (IV) administration of calcium gluconate or calcium chloride is crucial for patients suspected to have BRASH syndrome, as it helps stabilize the cardiac resting membrane potential, preventing cardiac arrhythmias and potential heart damage. While calcium chloride offers a threefold higher calcium concentration, the preferred option for peripheral delivery due to better patient tolerance is calcium gluconate. Typically, a dosage of 10 mL of a 10% solution or 1 gram of calcium gluconate is administered intravenously over five to 10 minutes, while for calcium chloride, the recommended dosage of 1 gram should be given over a slightly shorter period, about one to two minutes [7].

Combination medicines that promote potassium's entry into cells or boost its excretion from the body increase treatment response. For instance, insulin aids in the transport of potassium back into cells, which helps lower blood potassium levels. To prevent hypoglycemia, it is advised to first inject 10 units of normal insulin administered intravenously as a bolus, followed by 25 to 50 grams of 50% dextrose. Withholding dextrose when blood sugar levels are higher than 250 mg/dl has been suggested in certain studies, but it is vital to remember that insulin is eliminated through the kidneys. Therefore, avoiding dextrose in situations of acute renal damage may result in persistent hypoglycemia.

Another method for encouraging the transport of potassium into cells is nebulized albuterol. The typical dose for this therapy is 15-20 mg, and its efficacy is dose-dependent. Albuterol not only makes it easier for potassium to enter into cells, but it also causes sinus tachycardia as a positive side effect, which might be helpful in this clinical setting [8,9]. It is critical to focus on the root problem once the immediate life-threatening issues have been treated and handled. A patient's medical history by itself can frequently shed light on the trigger event. Moreover, as hypovolemia is the most frequent contributing factor, performing bedside restricted ultrasound tests is crucial to determine volume status. The patient's unique situation should be taken into account while administering IV hydration. Dialysis implications matter.

Remembering to consider the initial acid-base imbalance is crucial when using a pH-guided method during a large-volume resuscitation. Balanced crystalloids are a good option when the initial pH is between 7.35 and 7.45. It is important to note that hyperchloremic acidosis has been linked to prolonged usage of 0.9% normal saline. As a result, it is best to save this treatment for those who present with metabolic alkalosis [10].

A non-anion gap metabolic acidosis, more especially uremic acidosis, is commonly seen in patients with BRASH syndrome. As a result, it is suggested that the best fluid for therapy is isotonic bicarbonate, more particularly a solution containing 150 mEq/L of sodium bicarbonate in D5W. Using bicarbonate for resuscitation did appear to reduce mortality, according to the primary outcome, although this difference was not statistically significant. However, by lowering the requirement for urgent dialysis from 52% to 35% (p = 0.0009), the experiment did show a significant reduction in morbidity [11]. When hypotension does not improve after receiving IV fluids, vasopressor treatment must be started to improve organ perfusion. According to some research, beginning an isoproterenol or epinephrine infusion could be the best course of action in such circumstances [12].

This case highlights the importance of considering BRASH syndrome in patients with unexplained genital and lower extremity edema and underscores the need for a comprehensive and collaborative approach to manage the intricate medical issues associated with this condition. BRASH syndrome, while rare, should be recognized and considered in the differential diagnosis when encountering similar clinical presentations.

## Conclusions

This case report highlights the rarity and complexity of BRASH syndrome, characterized by bradycardia, renal dysfunction, AV nodal blockage, shock, and hyperkalemia. The interplay between AV nodal blockers and hyperkalemia can lead to life-threatening bradycardia, necessitating immediate interventions, such as calcium administration, insulin therapy, and albuterol nebulization. A multidisciplinary approach and increased awareness are crucial for early recognition and effective management in an aging population with evolving cardiovascular treatments. Further research is needed to better understand this syndrome and improve patient outcomes.
